# Generating synthetic identifiers to support development and evaluation of data linkage methods

**DOI:** 10.23889/ijpds.v9i5.2483

**Published:** 2024-09-18

**Authors:** Joseph Lam, Andy Boyd, Robin Linacre, Ruth Blackburn, Katie Harron

**Affiliations:** 1 Population, Policy & Practice Research and Teaching Department, UCL Great Ormond Street Institute of Child Health; 2 Population Health Sciences, Bristol Medical School, University of Bristol; 3 UK Ministry of Justice

We aimed to develop a framework for generating synthetic identifier datasets to support development and evaluation of data linkage methods. We evaluated whether replicating associations between attributes and identifiers improved the utility of the synthetic data for assessing linkage error.

We determined the steps required to generate synthetic identifiers that replicate the properties of real-world data collection. We generated synthetic versions of a large United Kingdom cohort study (the Avon Longitudinal Study of Parents and Children), according to the quality and completeness of identifiers recorded over several waves of the cohort. We evaluated the utility of the synthetic identifier data in terms of assessing linkage quality (false matches and missed matches).

Comparing data from two collection points in ALSPAC, we found within-person disagreement in identifiers (differences in recording due to both natural change and non-valid entries) in 18\% of surnames and 12\% of forenames. Rates of disagreement varied by maternal age and ethnic group. Synthetic data provided accurate estimates of linkage quality metrics compared with the original data (within 0.13-0.55\% for missed matches and 0.00-0.04\% for false matches). Incorporating associations between identifier errors and maternal age/ethnicity improved synthetic data utility.

Replicating dependencies between attribute values (e.g. ethnicity), values of identifiers (e.g. name), identifier disagreements (e.g. missing values, errors or changes over time), and their patterns and distribution structure enables generation of realistic synthetic data that can be used for robust evaluation of linkage methods. 

Our framework provides a novel and generalisable mechanism for developing and benchmarking record linkage algorithms.

